# Gradient Microstructure Evolution and High-Temperature Water Corrosion Behavior of Surface Mechanical Attrition Treated Alloy 600MA

**DOI:** 10.3390/nano16171080

**Published:** 2026-08-31

**Authors:** Jiahua Liu, Hongzeng Yang, Xinzhi Zhou, Maoping Wei, Yongming Han, Xiaofeng Zhang, Yonghao Lu, Andreas Rosenkranz, Xue Mi, Long Xin

**Affiliations:** 1 National Center for Materials Service Safety, University of Science and Technology Beijing, Beijing 100083, China; m202411232@xs.ustb.edu.cn (J.L.); 13105417783@163.com (H.Y.); zxz6491090@163.com (X.Z.); weimpqdu@163.com (M.W.); ymhan@ustb.edu.cn (Y.H.); zhangxiaofeng@ustb.edu.cn (X.Z.); lu_yonghao@mater.ustb.edu.cn (Y.L.); 2 Department of Chemical Engineering, Biotechnology and Materials, University of Chile, Santiago 8370415, Chile; arosenkranz@ing.uchile.cl; 3 National Key Laboratory of Nuclear Reactor Technology, Nuclear Power Institute of China, Chengdu 610213, China; xuefeinan@126.com

**Keywords:** SMAT, grain refinement, high-temperature water corrosion, Alloy 600MA

## Abstract

Gradient-nanostructured Alloy 600MA was fabricated via surface mechanical attrition treatment (SMAT) for 0–180 min. Pre-corrosion XRD confirms that SMAT with severe plastic deformation does not induce phase transformation of the γ-FCC Ni-Cr-Fe matrix, while progressive peak broadening verifies grain refinement and accumulated lattice strain, consistent with TEM-observed gradient nanolayers. Quantitative grain-boundary misorientation and mechanical-twinning information cannot be obtained solely from TEM-SAED. Pre-corrosion SEM-EDX reveals no obvious elemental segregation after SMAT. Corrosion behavior was evaluated in oxygen-saturated deionized water at 290 °C and 8.7 MPa for a single duration of 720 h. Raman spectroscopy detects crystalline corrosion products of NiCr_2_O_4_, though amorphous or disordered nanocrystalline oxides cannot be excluded. Under 720 h exposure, all SMAT-treated samples exhibit higher mass loss than the pristine alloy; the 120 min-SMAT sample shows moderate mass-loss among treated groups. Since defect-driven electrochemical activity and Cr diffusion were not directly measured, the competitive mechanism between defect-accelerated corrosion and Cr-enriched passivation is proposed based on experimental results and published literature. This work provides fundamental data for gradient-nanostructured nickel-based alloys under high-temperature pressurized-water conditions.

## 1. Introduction

With the rapid global development of nuclear power technology, pressurized water reactors (PWRs) have become the most widely used reactor type due to their high technical maturity and operational safety [1]. Steam generator (SG) tubes, as the key pressure boundary between the primary and secondary circuits of PWRs, serve in extremely harsh environments characterized by high temperature, high pressure, high flow rate and complex water chemistry conditions [2]. Their service reliability directly determines the safety and economic efficiency of nuclear power plants. Nickel-based Alloy 600 (nominal composition: Ni-15Cr-9Fe) has been extensively adopted as a SG tube material since the 1960s, owing to its excellent high-temperature mechanical strength, good thermal conductivity and moderate corrosion resistance in high-temperature aqueous environments [3].

However, field-service experience demonstrates that Alloy 600 components are susceptible to multiple forms of corrosion-related degradation, including uniform oxidation, pitting corrosion, intergranular corrosion and stress corrosion cracking (SCC) [4,5,6]. These degradation modes have caused multiple SG tube leakage accidents and forced shutdowns of nuclear power units worldwide. Statistical data show that PWSCC of Alloy 600 SG tubes is one of the most common failure modes in operating PWRs, resulting in huge maintenance costs and potential nuclear safety risks. Therefore, investigating the corrosion resistance of Alloy 600 in high-temperature pressurized water is of great engineering significance for extending the service life of nuclear power components and ensuring reactor safety.

To address the corrosion problem of Alloy 600, researchers have developed various modification approaches, including alloying optimization, heat treatment regulation and surface engineering [7]. Among them, surface modification technology has attracted extensive attention because it can optimize surface performance without changing bulk material properties, and has low cost and high flexibility. In recent years, surface nanocrystallization induced by severe plastic deformation has emerged as a promising surface-modification strategy. Different from traditional coating technologies, SPD-based surface nanocrystallization constructs a gradient nanostructured layer on the material surface through large plastic deformation, realizing metallurgical bonding between the modified layer and the matrix without interface-delamination risk [8].

Surface mechanical attrition treatment (SMAT) is a typical SPD surface nanocrystallization technique [9]. Its basic principle is to drive hundreds of hard ceramic balls to repeatedly impact the material surface at high speed under mechanical vibration, introducing severe plastic deformation into the surface layer and gradually refining coarse grains into nanoscale grains from the surface to the interior. Compared with traditional shot peening, SMAT can realize a higher strain rate and larger plastic deformation in the surface layer, and obtain thicker nanocrystalline layers with more uniform grain size distribution [10]. Compared with laser shock peening, SMAT has the advantages of simple equipment, low processing cost and suitability for complex-shaped components. Up to now, SMAT has been successfully applied to various metallic materials, including steels, aluminum alloys, titanium alloys and nickel-based alloys, and is known to substantially enhance surface hardness, wear resistance and fatigue performance [11,12,13].

In terms of corrosion performance, the effect of SMAT-induced nanocrystallization is complex and controversial, depending on the material system, corrosion environment and treatment process. For stainless steels, many studies have shown that SMAT can significantly improve corrosion resistance. Balusamy et al. found that SMAT of AISI 304 stainless steel promoted the formation of a more stable passive film in 3.5% NaCl solution, and pitting corrosion resistance was significantly enhanced [9]. They attributed this improvement to enhanced Cr diffusion through grain boundaries, which facilitated the rapid formation of a Cr-rich passive film. However, some studies also reported that SMAT reduced the corrosion resistance of stainless steels in acidic environments, because high-density defects increased surface electrochemical activity and accelerated anodic dissolution.

For nickel-based alloys, relevant research is still limited. Li et al. investigated the effect of surface nanocrystallization on the corrosion behavior of Alloy 690 in high-temperature water, and found that the nanocrystalline surface promoted the formation of a protective Cr-rich oxide scale [14]. However, Alloy 690 contains up to 30 wt.% Cr, while Alloy 600 only contains about 15 wt.% Cr, and their oxidation and corrosion behaviors differ significantly. For Alloy 600, most existing SMAT studies focus on mechanical properties and fatigue performance, while systematic research on the evolution of gradient microstructure with treatment time and its correlation with high-temperature water corrosion behavior is still insufficient. In particular, the competitive mechanism between defect-induced corrosion acceleration and diffusion-induced passivation enhancement during the SMAT process has not been clearly defined, and the intrinsic correlation between gradient microstructure characteristics and oxide scale formation dynamics remains to be revealed.

In this work, Alloy 600MA was subjected to SMAT with different durations (15, 45, 120 and 180 min). The gradient microstructure evolution with treatment time was systematically characterized by cross-sectional SEM and TEM, and the corrosion behavior in oxygen-saturated high-purity deionized water at 290 °C and 8.7 MPa was evaluated via weight-loss measurements. The morphology and composition of surface oxide scales were analyzed by SEM and Raman spectroscopy, and the competitive regulation mechanism of surface nanocrystallization on high-temperature water corrosion is discussed in depth. This work aims to reveal the microstructure–corrosion relationship of SMAT-modified Alloy 600, and provide a scientific basis for the surface modification design of nickel-based alloys serving in high-temperature aqueous environments.

## 2. Experimental

Alloy 600 mill-annealed (MA) plates with the following nominal chemical composition (wt.%): Ni balance, Fe 11.0, Cr 15.2, C 0.022, Ti 0.30, Mn 0.23, Si 0.29, P 0.0038, S 0.0025, were first machined into disk specimens with a diameter of 100 mm and a thickness of 4 mm. One flat circular surface of each disk was sequentially ground with 400#, 800#, 1200# and 2000# SiC abrasive papers, then mechanically polished using 2 μm diamond suspension on a velvet polishing cloth to obtain a mirror-smooth surface, followed by ultrasonic cleaning in ethanol for 10 min and drying with cold compressed air. SMAT (SNC-1, Chengu, China) was performed on the polished single surface of each disk at room temperature under 5 Pa in vacuum using 3 mm zirconia ceramic balls (Chengdu, China) and a 50 Hz vibration generator, with treatment times of 15, 45, 120, and 180 min. Only the targeted top surface was directly impacted by ceramic balls and received gradient nanocrystallization modification; the back surface and side edges of the disks remained unaffected by SMAT. After SMAT, small rectangular specimens with dimensions of 10 × 10 × 4 mm^3^ were wire-cut from the central uniform-deformation region of each SMAT-treated disk. The top surface of each small specimen retained the complete SMAT-modified layer, while the four side faces and the bottom face were non-SMAT-treated bulk matrix. All side and bottom surfaces were reground and repolished to the same finish as the initial pre-SMAT state, and ultrasonically cleaned before subsequent autoclave tests.

X-ray diffraction (XRD) measurements were carried out on all SMAT-treated Alloy 600MA specimens (0 min, 15 min, 45 min, 120 min, 180 min) prior to high-temperature water corrosion, to evaluate possible phase reorganization, phase transformation and microstructural change induced by severe plastic deformation. XRD data were collected using Cu-Kα radiation (λ = 0.15406). The scanning 2θ range was set from 10° to 90°, with a scanning rate of 2 °/min. Phase identification was performed by matching diffraction patterns with the PDF reference card. Peak-broadening features were qualitatively analyzed to reflect grain-refinement and accumulated micro-lattice strain caused by SMAT.

After SMAT, all specimens were ultrasonically cleaned in acetone for 10 min and absolute ethanol for another 10 min, and blow-dried with high-purity cold nitrogen gas prior to autoclave testing.

Three parallel replicate specimens were tested for each SMAT condition to ensure statistical reliability. Weight-change measurements were performed using an analytical electronic balance with a resolution of 0.01 mg.

Each specimen was machined to dimensions of 10 × 10 × 4 mm^3^. The total geometric surface area of each specimen was calculated to be 3.6 cm^2^, and all mass-loss data were normalized to this total surface area and reported in units of mg·cm^−2^. No masking material was applied; all surfaces of the specimen were directly exposed to the high-temperature aqueous environment. It should be noted that only the 10 × 10 mm^2^ top surface of each specimen possessed the SMAT-induced gradient nanostructure, while the four side faces and the bottom face were untreated bulk alloy with identical surface finish across all SMAT groups. Since the corrosion response of these non-SMAT surfaces is consistent among all specimen groups, the observed differences in total mass loss predominantly originate from the varied corrosion behavior of the SMAT-modified top surfaces, which ensures the validity of inter-group comparison. The reported normalized mass-loss values reflect the average corrosion response of the whole specimen.

After 720 h corrosion exposure, oxide scale removal was performed following a modified ASTM G1-03 standard practice. Specimens were immersed in boiling alkaline chromate solution (50 g NaOH + 25 g Na_2_CrO_4_ per 250 mL deionized water) for 5 min to dissolve surface corrosion oxide products. Immediately after boiling treatment, specimens were sequentially rinsed in hot deionized water, cold deionized water, and absolute ethanol, followed by ultrasonic cleaning in ethanol for 5 min, and blow-dried with high-purity nitrogen gas before gravimetric weighing.

Important note: According to ASTM G1-03, boiling alkaline chromate solution is designed to selectively remove corrosion oxides with minimal attack on Ni-base alloy substrates. Blank correction tests to quantify substrate dissolution originating from the oxide removal procedure were not performed in the present work. Therefore, the reported mass-loss values may contain minor contributions from substrate attack during the chemical-cleaning step.

High-temperature pressurized water corrosion experiments were carried out in a static autoclave under 290 °C, 8.7 MPa, in an oxygen-saturated aqueous environment, for a total corrosion exposure duration of 720 h. No boron or lithium species were intentionally added to the test medium. The test medium was high-purity deionized water. No external hydrogen gas was injected. Oxygen-saturated conditions were achieved via gas–liquid equilibrium inside the sealed static autoclave under the test temperature and pressure. No additional oxygen gas was bubbled into the water, and dissolved oxygen concentration was not continuously monitored during the 720 h static exposure. No external hydrogen gas was injected into the test medium. Dissolved hydrogen content was not measured or controlled during the static autoclave tests. The resistivity of starting deionized water was >15 MΩ·cm. Room-temperature pH of starting water was 6.8–7.0. Water aliquots were sampled before and after the 720 h corrosion test.

**Figure 1:**
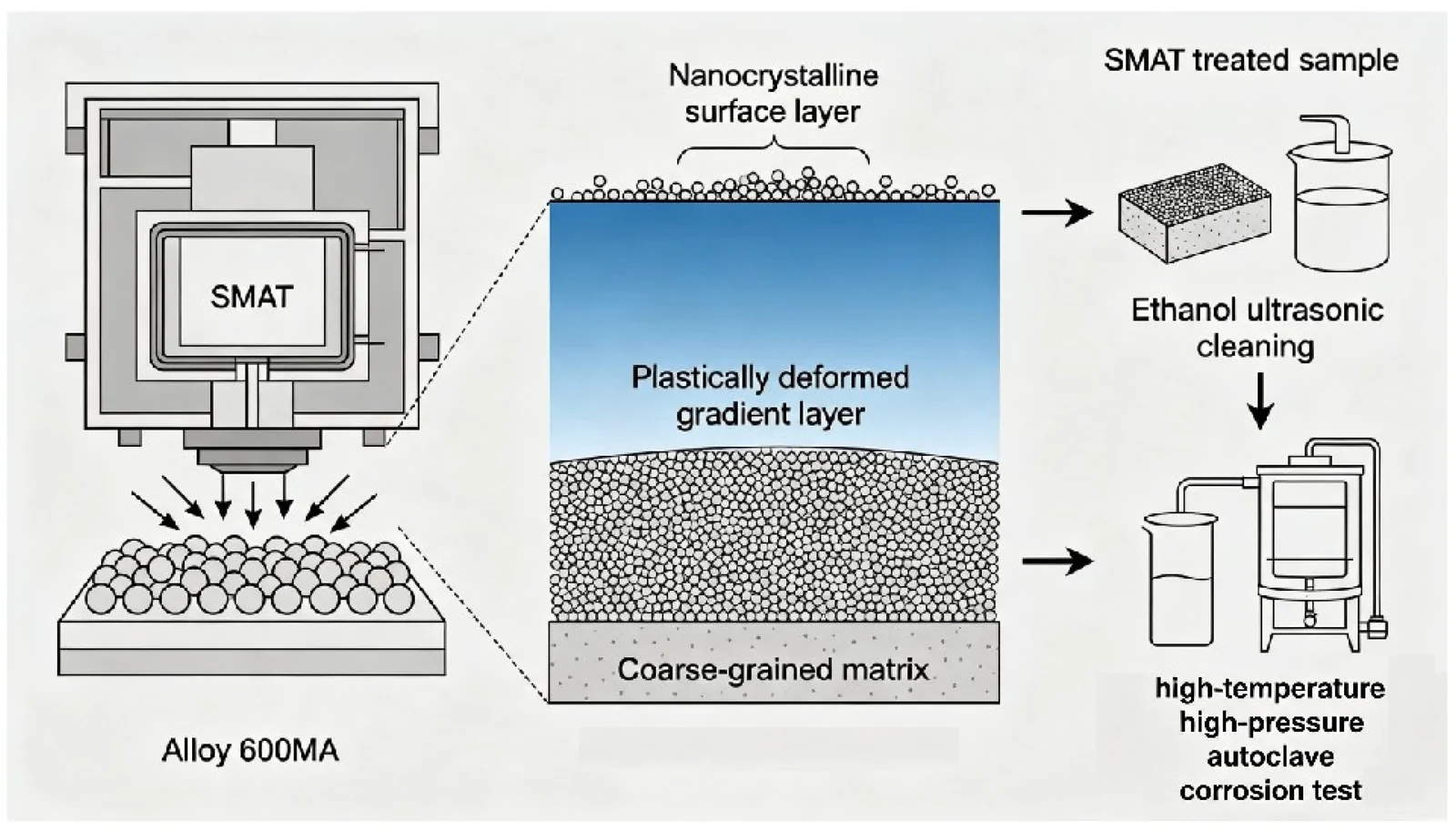
Schematic illustration of the SMAT process for Alloy 600MA, illustrating high-energy zirconia ball impact, the formation of gradient nanostructure including nanocrystalline surface layer, plastically deformed gradient layer and coarse-grained matrix, as well as subsequent sample ultrasonic cleaning and high-temperature pressurized-water autoclave corrosion test.

The complete workflow of SMAT and subsequent high-temperature pressurized water corrosion test is schematically illustrated in Figure 1. The SMAT process is similar to that reported in Ref. [15].

Cross-sectional gradient microstructures were characterized via scanning electron microscopy (SEM, ZEISS Auriga, Carl Zeiss AG, Oberkochen, Germany) equipped with an annular backscattered (AsB) electron detector. Imaging was performed at an accelerating voltage of 15 kV with a working distance of 6–8 mm. Thin TEM foils possessing a thickness of less than 70 nm were fabricated using the built-in focused ion beam (FIB) module of the same ZEISS Auriga dual-beam FIB-SEM system. Bright-field transmission electron microscopy (BFTEM) and selected area electron diffraction (SAED) characterizations were carried out on a Thermo Scientific Talos F200X S/TEM (Thermo Fisher Scientific, Hillsboro, OR, USA) operated at an accelerating voltage of 200 kV, equipped with a Schottky X-FEG electron source and Super-X windowless EDS detector.

Prior to high-temperature water corrosion testing, energy-dispersive X-ray spectroscopy (EDX) integrated with the ZEISS Auriga FIB-SEM was performed on SMAT-treated specimens (15, 45, 120, 180 min) and an untreated Alloy 600MA substrate to detect the initial surface elemental atomic distribution, without corrosion exposure. EDX area scanning was operated at 20 kV accelerating voltage, 8 mm working distance. For each sample, 5 random 5 × 5 μm^2^ surface regions were measured to obtain average atomic fractions of Ni, Cr, Fe, Ti, Mn, and Si, eliminating local statistical deviation.

Surface oxide phase analysis was conducted using a confocal micro-Raman spectrometer (inVia, Renishaw plc, Wotton-under-Edge, UK). A 532 nm solid-state green laser was selected as the excitation source, with a focused laser spot with a ~1 μm diameter via a 50× objective lens. The grating density was 1800 lines/mm, the spectral scanning range was set to 100–1200 cm^−1^, the laser power on the sample surface was strictly controlled at 2 mW to avoid laser-induced thermal degradation of NiCr_2_O_4_ spinel scale, and the integration time for each spectrum was 60 s. All Raman spectra were pre-calibrated with a standard silicon wafer (520.7 cm^−1^ characteristic peak) before testing to eliminate spectral offset errors.

## 3. Results and Discussion

### 3.1. Gradient Microstructure Evolution

**Figure 2:**
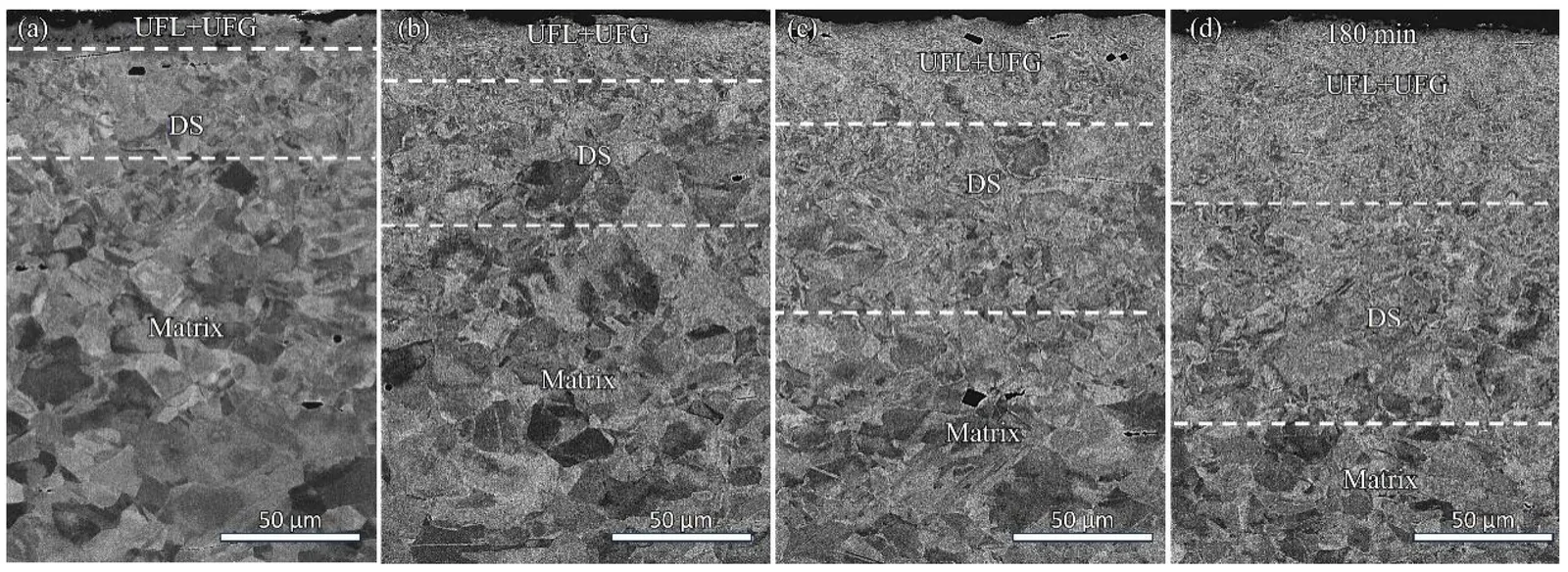
Cross-sectional SEM-AsB images of Alloy 600MA subjected to SMAT for (**a**) 15 min, (**b**) 45 min, (**c**) 120 min, and (**d**) 180 min, showing gradient microstructure evolution.

Cross-sectional SEM-AsB images of Alloy 600MA specimens with different SMAT durations are shown in Figure 2. It can be clearly observed that SMAT introduces a gradient-deformed layer on the specimen surface, and the total thickness of the deformed layer increases progressively with the extension of treatment time. From the surface to the matrix, the deformed layer presents obvious layered characteristics, which can be divided into two typical regions according to microstructure morphology: a top featureless nanocrystalline region and an underlying dislocation structure (DS) region. For the specimen treated for 15 min (Figure 2a), only a very thin continuous deformed layer is formed on the surface, with a thickness of only about 5–8 μm. The top layer is not completely featureless, and some deformed grain boundaries and slip traces can still be observed, indicating that the plastic deformation degree is relatively low at this stage, and grain refinement is insufficient. With the treatment time extended to 45 min (Figure 2b), the thickness of the deformed layer increases significantly, and a uniform featureless layer with a thickness of about 20 μm is formed on the top surface. Below the featureless layer, the DS region with obvious slip band characteristics can be observed, and the deformation degree gradually decreases with the increase in depth until it transitions to the undeformed matrix. When the treatment time reaches 120 min (Figure 2c), the thickness of the top featureless nanocrystalline layer further increases to about 50 μm, and the DS region also extends deeper into the matrix. Further extending the treatment time to 180 min (Figure 2d), the thickness of the nanocrystalline layer continues to increase slightly, but the growth rate slows down significantly, showing a tendency of deformation saturation.

This gradient structure is a typical feature of SPD surface treatment [13]. During SMAT, the high-speed impact of ceramic balls generates a strong stress wave propagating from the surface to the interior of the material. The stress amplitude decays exponentially with increasing depth, resulting in a gradient distribution of plastic strain and strain rate. The surface layer bears the largest plastic strain, driving full grain refinement down to nanoscale dimensions, while in the subsurface region, the strain is insufficient to form nanograins, and only a large number of dislocations and slip bands are introduced, forming the DS region.

**Figure 3:**
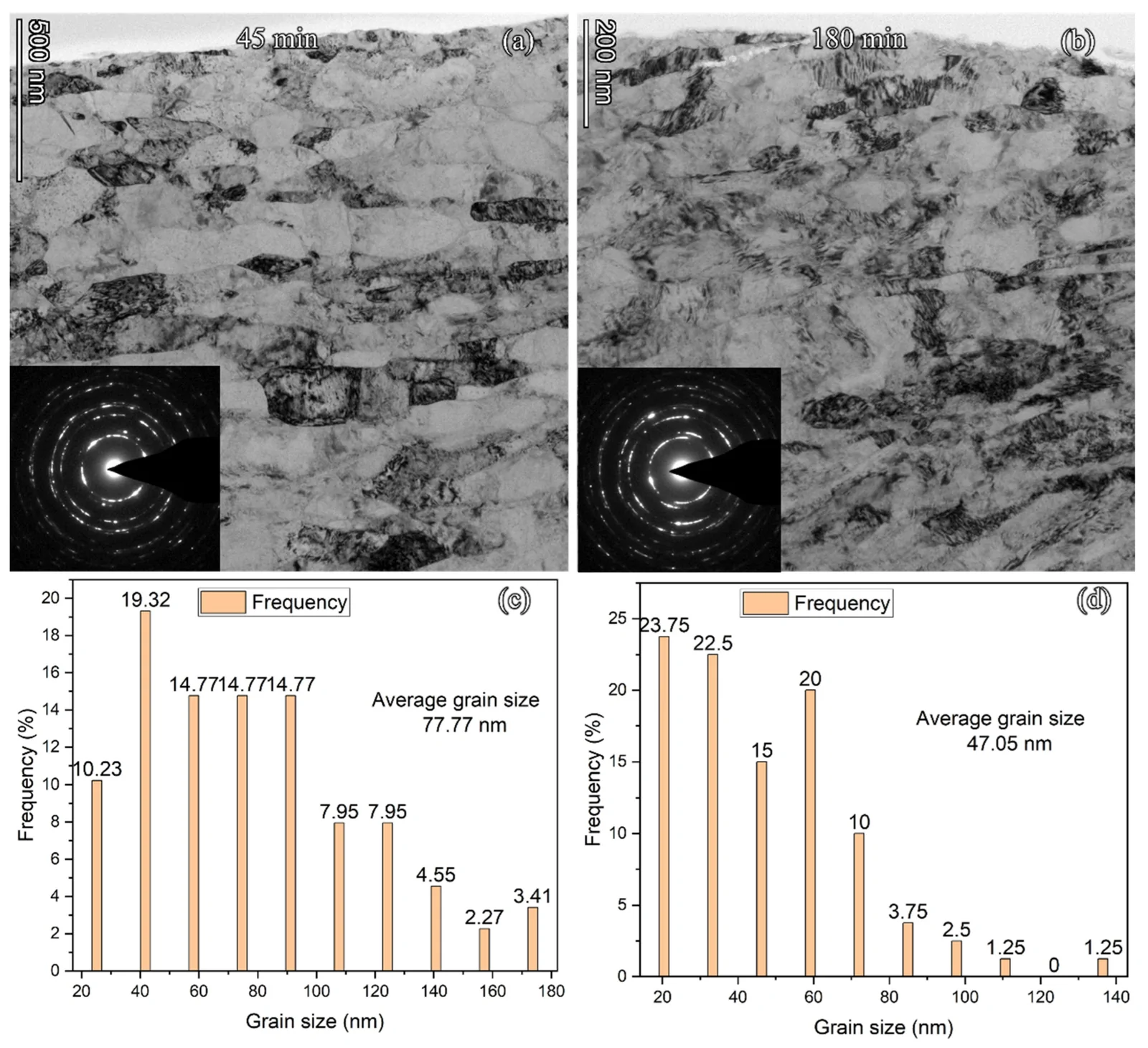
BFTEM micrographs and corresponding SAED patterns (insets) for samples treated at (**a**) 45 min and (**b**) 180 min. (**c**,**d**) Respective grain size distribution histograms.

To reveal the fine microstructure of the top nanocrystalline layer and its evolution with treatment time, TEM characterization was performed on 45 min and 180 min treated specimens, as shown in Figure 3. Both specimens show typical nanoscale microstructure characteristics, but there are significant differences in grain morphology and orientation. For the 45 min treated specimen (Figure 3a), the microstructure is dominated by elongated ultra-fine lamellae (UFL) mixed with a small amount of equiaxed ultra-fine grains (UFG). The grain boundaries are mostly low-angle boundaries formed by dislocation arrangement, and the SAED pattern (inset) shows discontinuous diffraction spots arranged in rings, indicating that grain orientation still has certain directionality and the proportion of high-angle grain boundaries is low. Statistical results show that the average grain size at 45 min is 77.77 nm, and the grain size distribution is relatively wide (Figure 3c). For the 180 min treated specimen (Figure 3b), the microstructure has completely transformed into uniform equiaxed nanograins with clear grain boundaries. The SAED pattern presents a continuous and uniform diffraction ring, which is widely interpreted in severe plastic deformation literature as an indicator of enhanced grain-orientation randomization and an increased fraction of high-angle grain boundaries. It should be noted that this is a qualitative inference supported by previous studies [16], rather than a direct quantitative measurement from SAED. The average grain size is refined to 47.05 nm, and the grain size distribution is more concentrated (Figure 3d). It is worth noting that although the treatment time is extended by four times from 45 min to 180 min, the average grain size is only reduced by about 39.5%, and the grain-refinement rate decreases significantly, which is a typical characteristic of deformation saturation in the SPD process.

Although TEM and SAED results confirm grain refinement and the formation of nanocrystalline layers after SMAT, these techniques alone cannot quantitatively characterize grain-boundary misorientation, grain-orientation distribution or identify mechanical deformation twins, since SAED only provides collective diffraction signals of multiple grains rather than local orientation information. Therefore, the increase in high-angle grain boundaries, randomization of grain orientation, activation of mechanical twinning, and the proposed sequence of grain refinement described below are literature-derived mechanistic possibilities [15,16], rather than direct quantitative experimental evidence obtained in the present work. According to previous studies on SMAT-processed face-centered cubic (FCC) nickel-based alloys, the grain refinement process generally proceeds via dislocation multiplication and rearrangement to form low-angle grain boundaries, followed by transformation into high-angle grain boundaries assisted by mechanical twinning, eventually producing randomly oriented nanograins in the near-surface region. Our cross-sectional TEM observations of grain size gradient are consistent with this literature-based grain-refinement scenario.

The grain-refinement process of Alloy 600 during SMAT can be explained based on the deformation mechanism of low stacking fault energy (SFE) FCC metals. The SFE of Alloy 600 is about 90–130 mJ/m^2^, which is a typical low-SFE material. For low-SFE FCC metals, the cross-slip of dislocations is difficult, and deformation is mainly dominated by planar slip and mechanical twinning. The grain-refinement process generally goes through the following stages:

1. Initial deformation stage: Under the repeated impact of ceramic balls, high-density dislocations are generated in the surface coarse grains. Since cross-slip is difficult, dislocations are arranged along specific slip planes to form dislocation walls and dislocation cells, dividing the original coarse grains into smaller sub-structures.
2. Lamellar structure formation stage: With the accumulation of plastic strain, mechanical twinning occurs in the deformed grains. A large number of nano-twins are generated and intersect with each other, further cutting the sub-structures into ultra-fine lamellae. At this stage, the boundaries are mainly low-angle grain boundaries and twin boundaries, corresponding to the microstructure characteristics of the 45 min specimen.
3. Equiaxed nanograin formation stage: With further increase in strain, continuous dislocation slip and grain rotation occur inside the lamellae, and the orientation difference between adjacent lamellae gradually increases. Low-angle boundaries gradually evolve into high-angle grain boundaries, and the lamellar structure is finally transformed into equiaxed nanograins with random orientations, corresponding to the microstructure characteristics of the 180 min specimen.

When the grain size is refined to a certain extent, the strength of the nanocrystalline layer increases significantly due to the fine-grain strengthening effect. Higher stress is required to further deform the nanograins, but the impact energy provided by SMAT is limited, so the grain-refinement rate gradually decreases and approaches a saturation state. This deformation saturation phenomenon is universal in the SMAT process and has been widely reported in various metal materials [17].

**Figure 4:**
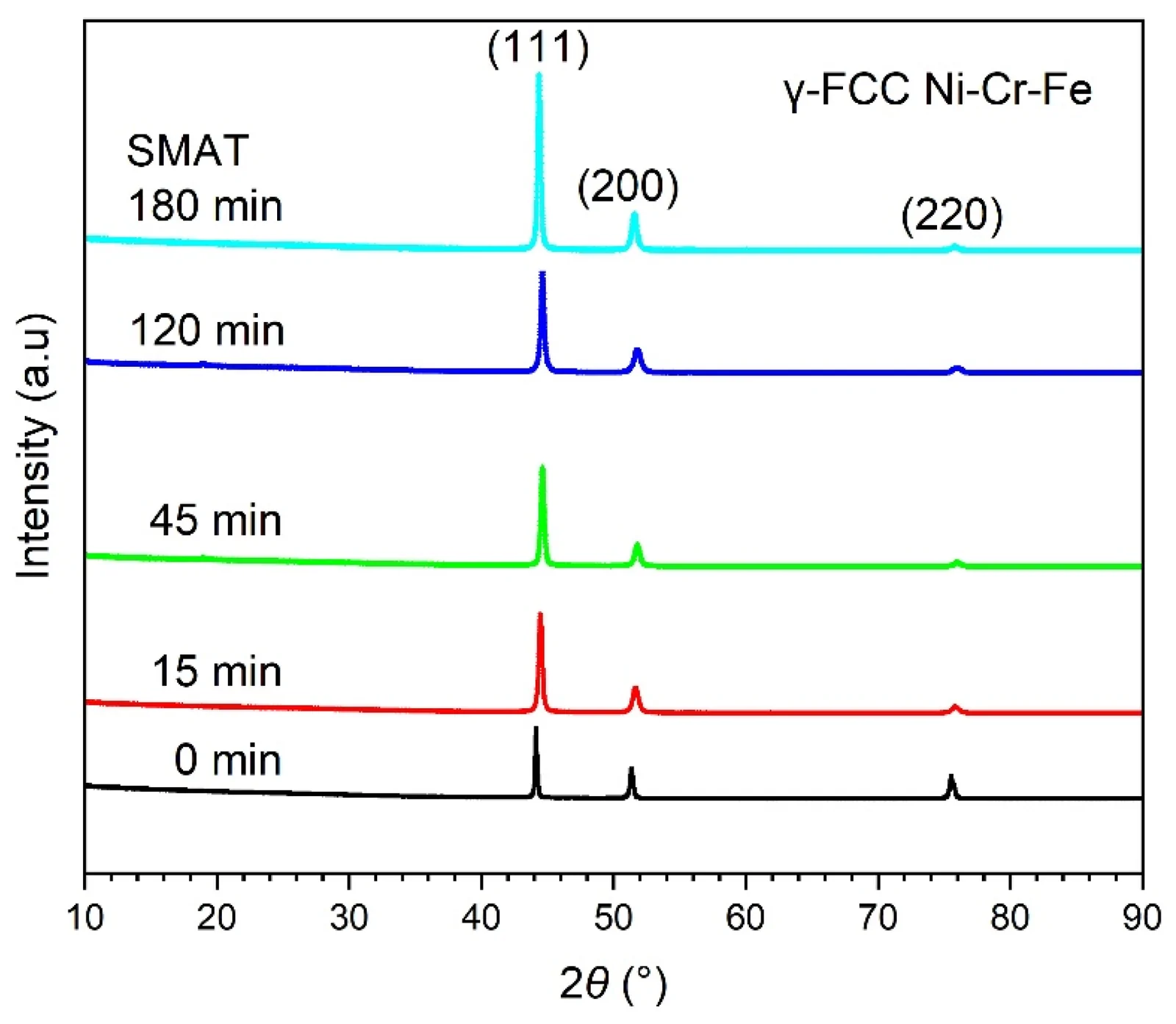
XRD patterns of Alloy 600MA after different SMAT durations (0 min, 15 min, 45 min, 120 min, 180 min). All measurements were carried out before autoclave corrosion tests. Peaks at ~44°, ~51°, ~76° are indexed to (111), (200), (220) planes of γ-FCC Ni-Cr-Fe solid-solution matrix.

Figure 4 displays XRD patterns of Alloy 600MA specimens subjected to different SMAT durations (0, 15, 45, 120, 180 min) prior to high-temperature water corrosion. Three characteristic diffraction peaks located near 2θ ≈ 44°, 51°, and 76° correspond to the (111), (200), and (220) crystallographic planes of the γ-FCC Ni-Cr-Fe solid-solution matrix (PDF No. 04-0850). No additional diffraction peaks belonging to oxides, carbides or intermetallic secondary phases are observed even for the longest 180 min SMAT. This confirms that severe plastic deformation introduced by SMAT does not trigger phase transformation or phase reorganization within Alloy 600MA. Nevertheless, obvious peak broadening occurs with increasing SMAT duration, originating from combined contributions of grain refinement and accumulated micro-lattice strain induced by severe plastic deformation. This observation is consistent with the gradient nanocrystalline surface layers revealed by TEM. It should be emphasized that these XRD measurements were performed before corrosion exposure. Thin oxide scales formed after high-temperature water corrosion cannot be distinguished by laboratory XRD due to overwhelming substrate diffraction signals; oxide-phase identification for corroded samples is therefore realized by Raman spectroscopy in this work.

### 3.2. Surface Oxide Evolution

**Figure 5:**
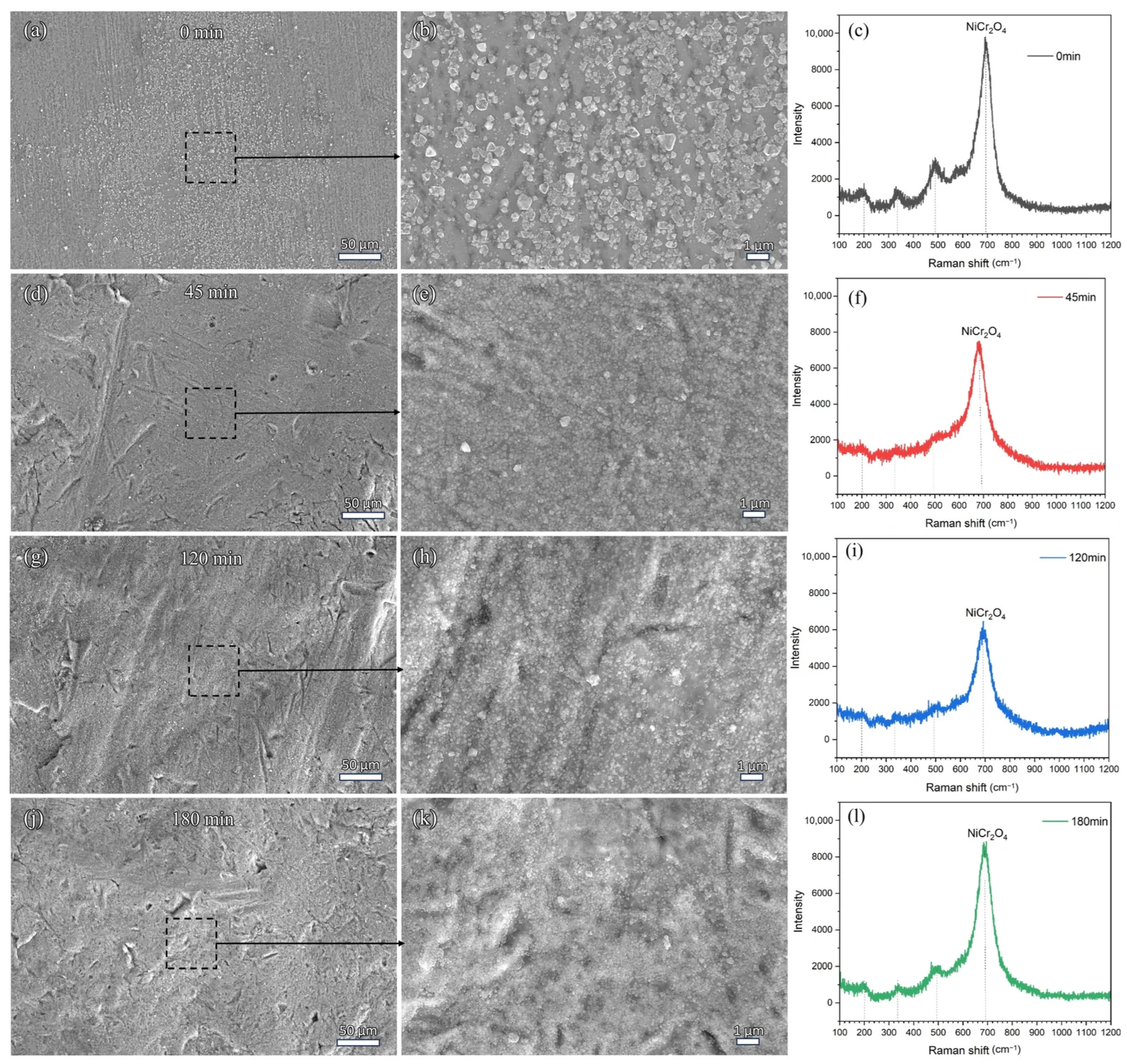
SEM micrographs and Raman analyses of surface oxides after corrosion: (**a**–**c**) untreated, (**d**–**f**) 45 min, (**g**–**i**) 120 min, (**j**–**l**) 180 min. Left: low magnification; middle: high magnification; right: Raman spectra.

The surface morphology of oxide scales formed on specimens with different SMAT durations after 720 h high-temperature water exposure is shown in Figure 5. Distinct differences in oxide morphology are observed across different SMAT durations, reflecting the important influence of surface microstructure on oxidation behavior. For the untreated specimen (Figure 5a,b), the surface oxide particles are sparsely distributed, with large size and irregular shape. The oxide particles are coarse and discrete, and a large area of the matrix can still be seen between the particles, indicating that the oxide scale is discontinuous and incomplete. This is because the surface grain size of the untreated specimen is large, the density of grain boundaries and defects is low, and the number of heterogeneous nucleation sites for oxidation is small. The oxide nuclei grow up gradually at limited nucleation sites, eventually forming coarse and discrete oxide particles. For the 45 min treated specimen (Figure 5d,e), the size of oxide particles is significantly refined, and the number density is greatly increased. The oxide particles are uniformly distributed on the surface, but they are still independent of each other and have not yet formed a completely continuous oxide scale. This is because SMAT introduces a large number of grain boundaries and defects to the surface, which greatly increases the heterogeneous nucleation sites of oxidation. According to classical nucleation theory, the nucleation rate of oxide is proportional to the density of nucleation sites. Therefore, the nucleation rate of oxide on the nanocrystalline surface is much higher than that on the coarse grain surface. Under the same oxidation time, a large number of fine oxide nuclei are formed, showing the characteristic grain refinement of oxidation products. For the 120 min and 180 min treated specimens (Figure 5g,h,j,k), the oxide particles further grow and connect with each other, forming a compact, continuous and uniform oxide scale on the whole surface. No exposed matrix can be observed, and the oxide scale presents a fine-grained dense morphology. With the extension of SMAT time, the compactness of the oxide scale further improves. This indicates that with the increase in grain-refinement degree, it is hypothesized that grain boundary short-circuit paths may promote outward Cr atom migration, favoring Cr-selective oxidation and oxide-scale growth, potentially yielding a continuous protective oxide film.

Raman spectroscopy was used to analyze the phase composition of oxide scales on different specimens, and the results are shown in Figure 5c,f,i,l. All specimens show four characteristic Raman peaks located at about 220 cm^−1^, 340 cm^−1^, 500 cm^−1^ and 690 cm^−1^, which are consistent with the standard Raman characteristic peaks of NiCr_2_O_4_ spinel phase [18]. No characteristic peaks of NiO, Cr_2_O_3_ or other nickel–iron–chromium oxides are detected, indicating that the NiCr_2_O_4_ spinel constitutes the dominant Raman-detectable crystalline phase on corroded specimen surfaces. It should be noted that minor amorphous or poorly crystallized phases cannot be excluded solely from Raman results. Although the phase composition is the same, the peak intensity and peak width of Raman spectra show obvious evolution with SMAT time. For the untreated specimen, the Raman peaks are sharp and high in intensity, indicating that the oxide has good crystallinity and large grain size. For the 45 min treated specimen, the peak intensity decreases significantly and the peak width increases, which corresponds to the fine-grained and low-crystallinity oxide morphology observed by SEM. For the 120 min and 180 min specimens, Raman peak intensity increases again and gradually exceeds that of the untreated specimen. This trend is consistent with improved oxide crystallinity and increased oxide-scale thickness with prolonged SMAT; however, Raman peak intensity alone cannot serve as direct quantitative evidence for oxide thickness, as it is also influenced by crystallite size, crystal orientation, and surface roughness. NiCr_2_O_4_ is a typical inverse spinel structure with a compact cubic lattice. Compared with NiO and other simple oxides, NiCr_2_O_4_ spinel has a lower cation diffusion coefficient and better chemical stability in high-temperature aqueous environment, and is recognized as the main protective oxide phase of nickel-chromium alloys in high-temperature water. The formation of a continuous NiCr_2_O_4_ scale can effectively block the inward diffusion of oxidizing species (such as O^2−^, OH^−^) and the outward diffusion of metal cations, thus inhibiting further corrosion of the matrix. Therefore, the densification and continuity improvement of the NiCr_2_O_4_ scale induced by SMAT modifies the surface oxidation behavior of Alloy 600MA under the tested conditions.

No characteristic Raman peaks assignable to crystalline are detected across all specimens. It should be emphasized that the absence of a Raman signal does not equate to complete absence of Cr_2_O_3_; minor Cr_2_O_3_ may exist in amorphous form, at concentrations below the Raman detection limit, or as a thin buried inner layer undetectable by surface-sensitive Raman measurement. According to published high-temperature water corrosion literature for nickel–chromium alloys, crystalline Cr_2_O_3_ is susceptible to dissolution and hydrolysis in high-temperature aqueous environments, forming soluble species such as Cr(OH)_3_ or HCrO_2_^−^, which limits its long-term stability as a crystalline surface phase. In contrast, crystalline NiCr_2_O_4_ spinel exhibits higher resistance to dissolution and is widely identified as the dominant detectable crystalline protective oxide phase for nickel–chromium alloys in high-temperature pressurized water.

The untreated substrate and all SMAT specimens (15–180 min) exhibited nearly identical atomic percentages of Ni, Cr and Fe on the surface, consistent with the nominal bulk composition of Alloy 600MA, as shown in Table 1. No obvious Cr enrichment or Fe depletion was observed on the surface of any SMAT-treated sample, even for the 180 min specimen with fully equiaxed nanograins. This result confirms that SMAT-induced severe plastic deformation and grain refinement do not cause measurable pre-segregation of Cr, Ni or Fe on the outermost surface at room temperature, which excludes pre-existing elemental segregation as a confounding factor for subsequent corrosion behavior. It should be clarified that pre-corrosion EDX results cannot directly demonstrate Cr diffusion behavior during the high-temperature corrosion process. The hypothesis of Cr outward diffusion along nanocrystalline grain boundaries during corrosion is supported by published SMAT corrosion literature [9,14], rather than being directly validated by the present EDX measurements.

Raman spectroscopy is only sensitive to Raman-active crystalline oxide phases. Therefore, the existence of thin amorphous layers, highly disordered nanocrystalline oxides, or buried chromium-rich inner oxide layers cannot be excluded based solely on the present Raman results. In this work, the observed Raman-shift signals can be assigned to crystalline NiCr_2_O_4_ corrosion products according to published reference spectra [18]. It should be emphasized that these represent only the detectable crystalline Raman-active fractions within the oxide scale. Minor amorphous or disordered nanoscale oxide constituents may co-exist but remain undetectable under the current measurement conditions. Further complementary characterizations including cross-sectional TEM and XPS will be required in future work to fully resolve the complete phase constitution and elemental distribution of the multi-layer oxide scale.

Raman spectroscopy is only sensitive to surface-accessible, Raman-active crystalline oxide phases. Therefore, conclusions regarding oxide phase constitution in this work are strictly limited to crystalline fractions detectable by Raman measurement. The existence of thin amorphous layers, highly disordered nanocrystalline oxides, or buried chromium-rich inner oxide layers cannot be excluded based solely on the present Raman results.

### 3.3. Corrosion Behavior and Mechanism

It should be emphasized that defect-driven electrochemical activity and chromium diffusion kinetics are not directly quantified or measured in the present study. The following discussion contains both experimentally measured observations and mechanistic interpretations inferred from our experimental phenomena combined with previously published literature. Experimental facts include gravimetric corrosion mass-loss data, cross-sectional grain-size gradient from TEM, pre-corrosion EDX elemental analysis and Raman-detected crystalline corrosion products. In contrast, statements regarding defect-enhanced electrochemical activity and grain-boundary accelerated Cr outward diffusion represent proposed mechanistic explanations rather than direct experimental measurements obtained in this work [11,15,17,19,20]. It is hypothesized, and explicitly supported by extensive published SMAT corrosion studies [9,14], that SMAT-generated high-density grain boundaries act as short-circuit diffusion channels and promote outward Cr migration. This mechanistic interpretation is not directly validated by experimental measurements in the present work.

**Figure 6:**
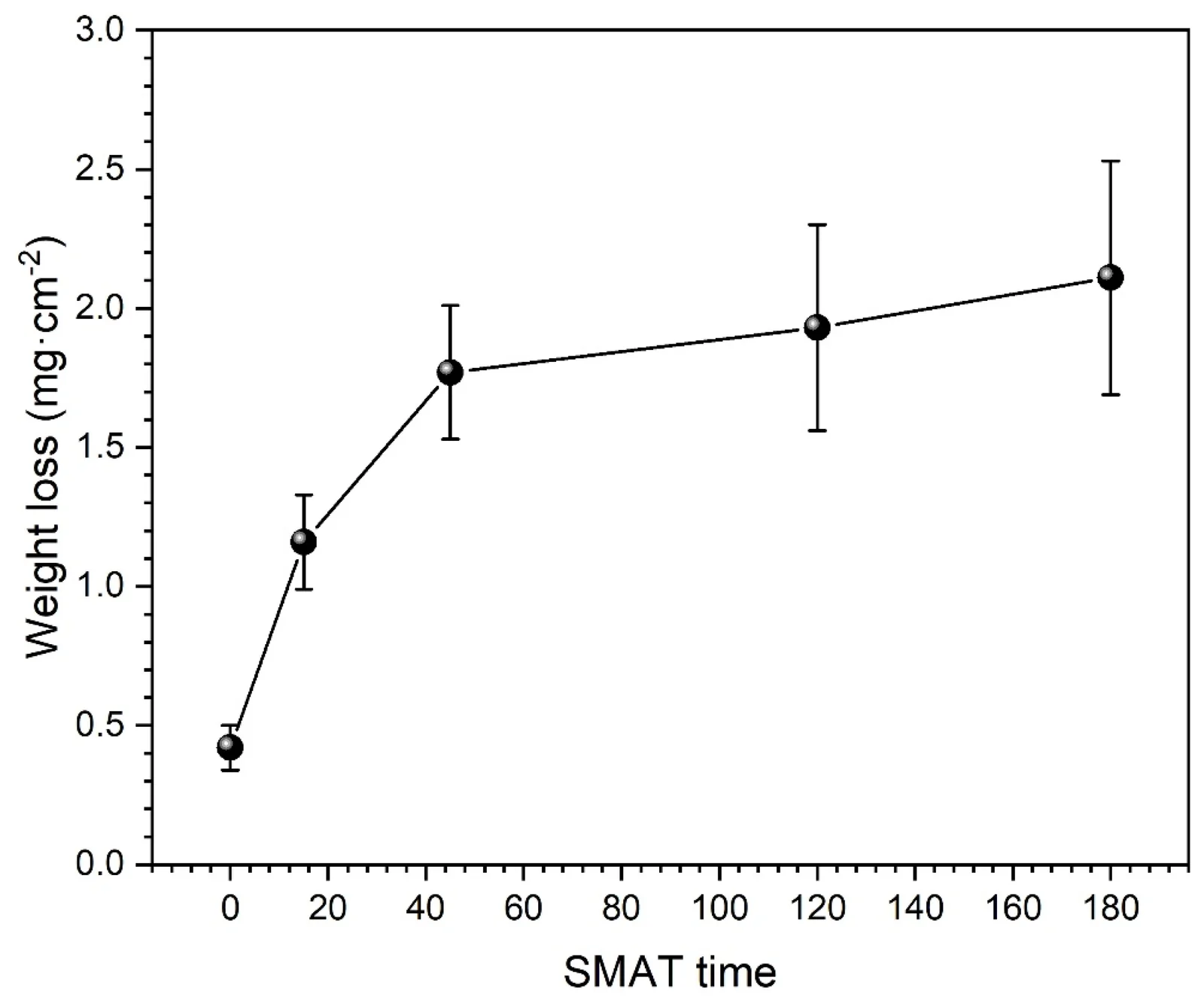
Normalized corrosion mass-loss of Alloy 600MA as a function of SMAT duration after 720 h exposure. Mass loss values are normalized to the total geometric surface area of each specimen, with units of mg·cm^−2^. Error bars denote standard deviation obtained from three replicate samples per condition.

The corrosion weight loss of Alloy 600MA specimens with different SMAT durations after 720 h high-temperature water exposure is shown in Figure 6. The weight loss presents a very distinct two-stage evolution trend with the extension of SMAT time:

(1) Rapid increase stage (0–45 min): The normalized mass loss of the untreated specimen is only 0.42 mg·cm^−2^, while that of the 45 min treated specimen rises sharply to 1.77 mg·cm^−2^, with an increase of 321%. This indicates that under the tested 720 h condition, surface nanocrystallization induced by short-time SMAT accelerates the material mass loss rate in high-temperature water.
(2) Slow-rising level-off stage (45–180 min): After 45 min, the rate of mass-loss growth slows markedly. The normalized mass loss at 120 min reaches 1.93 mg·cm^−2^, only 9.0% higher than that at 45 min; the value at 180 min is 2.11 mg·cm^−2^, only 9.8% higher than that at 120 min. The growth of mass loss slows markedly and the curve levels-off with further increasing SMAT duration, under the fixed 720 h corrosion-exposure condition. This indicates that with the further extension of SMAT time, the increase in mass loss becomes limited within the 720 h exposure window.

In addition, the error bars of weight loss at 120 min and 180 min are slightly larger than those at short treatment time, which may be due to the severe plastic deformation on the surface, resulting in local differences in surface roughness and deformation uniformity, which further leads to slight differences in the integrity of the oxide scale at different positions.

The unique bi-phasic corrosion behavior is essentially the result of the competition between two opposite effects induced by surface nanocrystallization: the “defect-activation effect” that accelerates corrosion and the “diffusion-passivation effect” that inhibits corrosion. The relative strength of the two effects changes with the extension of SMAT time, leading to the staged evolution of corrosion performance.

In the early stage of SMAT (0–45 min), the defect-activation effect plays a dominant role, leading to accelerated corrosion. SMAT introduces a large number of crystal defects such as grain boundaries, dislocations and vacancies into the surface layer. According to electrochemical corrosion theory, atoms at grain boundaries and defect positions have higher energy and higher electrochemical activity than those in the crystal interior, and are preferentially dissolved as anodic regions in the corrosion process. The higher the defect density is, the more anodic active sites are on the surface, and the higher the initial corrosion rate is. On the other hand, at this stage, the grain refinement degree is limited, and the Cr diffusion rate is not enough to form a complete protective oxide scale in a short time. The fine and discrete oxide particles cannot effectively block the corrosion medium, so the corrosion rate increases rapidly with the increase in defect density.

In the later stage of SMAT (45–180 min), the diffusion-passivation effect gradually occupies the dominant position, and the corrosion rate tends to be stable. With the extension of treatment time, the surface grain size is further refined, and the density of high-angle grain boundaries increases significantly. Grain boundaries are typical short-circuit diffusion channels, and the diffusion coefficient of atoms along grain boundaries is 10^4^–10^6^ times higher than that in crystal interior at room temperature, and still maintains a significant advantage at high temperatures. The high-density grain boundaries in the nanocrystalline layer greatly accelerate the outward diffusion rate of Cr atoms from the matrix to the surface. Sufficient Cr supply promotes the selective oxidation of Cr on the surface and the growth of NiCr_2_O_4_ spinel, and finally forms a dense, continuous and well-adhered protective oxide scale. Once the complete oxide scale is formed, it acts as an effective diffusion barrier, greatly reducing the transmission rate of corrosive media and metal ions, thus inhibiting further corrosion. At this stage, although the defect density continues to increase, the barrier effect of the oxide scale plays a more dominant role, so mass-loss no longer rises appreciably and levels-off under the fixed 720 h exposure window. It is emphasized that this trend describes mass-loss variation with SMAT duration at a fixed corrosion exposure time, and does not represent a kinetic plateau of corrosion rate as a function of extended exposure time.

This competitive mechanism is consistent with the research results of SMAT on other corrosion systems. For example, Balusamy et al. found that the corrosion current density of 304 stainless steel first increased and then decreased with the extension of SMAT time in NaCl solution, which also originated from the competition between defect activation and passivation enhancement. The difference is that Alloy 600 has a lower Cr content, so it takes longer SMAT time to form a complete protective oxide scale, and the plateau stage appears later.

It should be emphasized that all corrosion observations in this work are limited to the single investigated exposure duration of 720 h. We cannot draw conclusions about material behavior over extended service durations. Within our test window, SMAT-treated specimens exhibit higher mass-loss values compared with the untreated reference sample, accompanied by the formation of denser and more continuous NiCr_2_O_4_ oxide scales. Whether such oxide-scale features translate to altered corrosion performance for much longer exposure times remains unknown and requires multi-time-point corrosion experiments for further verification.

The gradient microstructure produced by SMAT alters surface defect states and grain-boundary density, and consequently modifies oxide-scale nucleation and growth under high-temperature-water conditions. The gradual microstructural transition from nanograined surface to coarse-grained matrix may mitigate sharp interfacial stress, as reported in existing gradient-structure literature. The subsurface dislocation-rich region may potentially serve as Cr diffusion paths to supply Cr for surface oxide growth; however, Cr diffusion kinetics were not directly measured in the present study. It should be noted that mechanical property evaluation and SCC tests were not performed in this investigation; therefore, no claims regarding mechanical performance or SCC susceptibility of SMAT-treated Alloy 600MA are made in this manuscript.

It is acknowledged as a methodological limitation that blank-correction tests for the oxide-removal procedure were not performed in this work. As noted in the Experimental section, the boiling alkaline chromate solution causes minimal attack on nickel-base alloy substrates according to ASTM G1-03, but minor substrate dissolution during chemical cleaning cannot be fully excluded. Therefore, the reported mass-loss values may contain a small systematic bias from substrate attack during oxide removal.

## 4. Conclusions

In this work, gradient-nanostructured layers with distinct microstructural features were produced on an Alloy 600MA surface by SMAT with different durations. The gradient microstructure evolution, oxide scale formation behavior and high-temperature water corrosion mechanism were systematically studied, and the main conclusions are as follows:

(1) SMAT successfully produces a typical gradient microstructure on Alloy 600MA surfaces, consisting of an outermost ultrafine-grained nanocrystalline layer and a subsurface dislocation-dominated deformation zone. As SMAT time increases, the nanocrystalline layer thickness gradually rises and reaches approximately 50 μm at 120 min; the average surface grain size decreases from 77.77 nm (45 min) to 47.05 nm (180 min). Microstructure evolves toward equiaxed nanograins, following the well-established grain-refinement mechanism for low-SFE FCC metals: dislocation slip → mechanical twinning → lamella subdivision → equiaxed nanograins, with the refinement rate gradually declining upon deformation saturation. It should be noted that high-misorientation grain boundaries and mechanical twinning are inferred based on published literature, since quantitative misorientation data are not available from the present TEM-SAED results.
(2) After 720 h high-temperature water-corrosion exposure, Raman-detectable crystalline corrosion product is dominated by NiCr_2_O_4_ spinel. SMAT does not alter the detectable oxide phase constitution, yet remarkably modifies oxide-scale morphology and compactness. With prolonged SMAT time, oxides evolve from coarse discrete particles into fine, dense continuous scales. Higher grain-boundary density originating from SMAT is hypothesized to accelerate oxide nucleation, while enhanced Cr outward grain-boundary short-circuit diffusion (not directly measured in this work) is proposed to facilitate scale growth and densification.
(3) The corrosion mass loss exhibits a two-stage trend versus SMAT duration under the fixed 720 h corrosion-exposure condition: a rapid rise within 0–45 min, followed by a slow-rising level-off from 45 min to 180 min. This trend is interpreted as a competitive effect induced by surface nanocrystallization. At early stages, high-density deformation defects increase surface reactivity and accelerate initial corrosion. At later stages, grain-boundary-accelerated Cr diffusion is presumed to favor the formation of compact protective NiCr_2_O_4_ scale and mitigate further corrosion. This interpretation remains a mechanistic hypothesis, as defect-driven activity and Cr-diffusion kinetics were not directly quantified experimentally.

## Figures and Tables

**Table 1:** Average atomic percentage (at.%) of major metallic elements on sample surfaces after SMAT (before high-temperature water corrosion).

SMAT Time (min)	Ni	Cr	Fe	Ti	Mn	Si
0 (untreated)	73.4	14.9	10.8	0.32	0.24	0.33
15	72.8	15.3	10.9	0.31	0.25	0.34
45	73.5	14.8	10.7	0.32	0.23	0.35
120	73.3	15.0	10.8	0.32	0.24	0.33
180	72.9	15.4	10.9	0.31	0.25	0.32

## Data Availability

The original contributions presented in this study are included in the article. Further inquiries can be directed to the corresponding author.
